# The governance of energy transitions in Africa: a sketch of plural perspectives

**DOI:** 10.1186/s13705-022-00380-2

**Published:** 2022-12-28

**Authors:** Philipp Späth, Vanesa Castán Broto, Simon Bawakyillenuo, Michael Pregernig

**Affiliations:** 1 University of Freiburg, Freiburg, Germany; 2 University of Sheffield, Sheffield, England; 3 University of Ghana, Accra, Ghana

## Abstract

Building on the contributions to the article collection “The Governance of Sustainable Energy Transitions in the Global South “, this editorial offers a sketch for a research agenda on transitions research with a main focus on Africa. Still being ill-defined in its concrete contours, this research agenda engages with the central themes of heterogeneity, politics, and the material basis of energy transitions. In this editorial, we address both procedural and content-related questions. Regarding procedural questions, we inform about the context in which this collection emerged. On that, a workshop held in Accra in September 2019 was a key milestone. We contextualise the challenges that some workshop participants had with developing their contributions into publishable articles in the context of uneven academic support structures and knowledge hegemonies. Finally, we introduce the contributions to our article collection, emphasising how they connect and contribute to our draft research agenda. With regard to the content dimension, this article collection builds and proclaims the need for plural approaches to understanding energy transitions in Africa. A plurality of specific context conditions calls for pluralistic analytical perspectives. Not taking for granted hegemonic, western ways of understanding energy systems and explaining change, we rather depart from engagements with the diversity of changes that aggregate into transition pathways—a diversity that in the context of Africa is impossible to overlook. To implement such a pluralistic research agenda, scholars need more opportunities to network, exchange and publish.

## Introduction

The 2020 World Energy Outlook examines the prospects for accelerated energy transitions away from fossil fuels (especially oil and coal) [1]. Similarly, the Africa Energy Outlook 2022 stipulates that “energy efficiency and renewables—especially solar—are key pillars for building Africa’s new energy economy” ([2]; p. 4). The reports define the energy transition as a structural transformation of the energy sector that requires capital for new infrastructures and efficiency measures. While this energy transition varies in pace, scale, and objectives across different geographies, there is an increasing tendency to think about it as a global transition [3]. It is global, both because of the magnitude of change required—whose impacts will likely reach all the confines of the World—and because it has constituted a standard discourse of energy governance, which is now ubiquitous in every geography. The energy transition, however, also results from the complex integration of highly globalized value chains and local material and institutional conditions that determine actual outcomes [4].

In Africa, the energy transition is interlinked with complex energy access challenges [5]. It has emphasized the need to draw attention to the productive sector as an engine for change [6]. Most studies of energy transitions in Africa emphasize the need to mobilize a wide array of actors operating at different scales. For some time, the idea of leapfrogging towards a more sustainable, renewable future dominated the debates on energy transitions in Africa [7]. Unlocking the transition is often seen as a question of overcoming policy and investment barriers to unlock the enormous potential of ‘indigenous sources of energy’ that could put Africa at the forefront of sustainability [8, 9]. In these accounts, human capital and capacity building appear as the keys to unlocking the transition [7, 8], echoing regional development approaches.

However, recent work has emphasized the necessarily fragmented nature of energy transitions and the need to foreground specific local conditions in understanding differentiated transition pathways [10]. There are continued demands for further investments in renewable energy and a sense that the gap is not being addressed as fast as it is desired in international organizations [11, 12]. At the same time, this investment gap is attributable to several binding constraints at different levels of intervention [13, 14]. Therefore, while the global nature of the challenge and some critical dynamics are widely acknowledged, so is the fact that essential context conditions and challenges for energy transitions differ substantially from country to country and from place to place. Such pluralities of material constraints and transition pathways are not consistently recognized in the dominant literature that often assimilates idealized conditions for transition (with particular reference to the case of South Africa) to a whole continent.

Moreover, the last decade has seen an increasing interest in understanding the conditions of transition because, as explained by geographer Cheryl McEwan, transitions are not only bound up with material constraints related to the technologies, resources, and land available but also with complex historical trajectories and complex politics, which in the African context are often impossible to extricate from their colonial histories [15]. One of the most productive areas of study in understanding energy transitions in Africa has therefore been to engage with the political economy of energy in different countries. This can be fruitful from an analytical perspective—examining e.g. how economic barriers to the transition are intertwined with political dynamics—or from critical ones—for example examining how the political-economic configurations of power reproduce existing inequalities and injustices within the energy system. In most cases, these perspectives are actually combined (e.g., [12, 16–20]). What emerges as an alternative to these perspectives is an engagement with the place-based practices in which different transition alternatives are explored, for example by constituting ‘experimental zones’ [15]. In several cases, academics have become embedded in making such experimental zones through locally-based co-design experiments that herald a different approach to energy transitions [21]. Often, modular, decentralized systems offer opportunities for the constitution of such experimental zones [22].

In his latest book, Swilling observes two tendencies in responding to the need for sustainability transitions, of which energy transitions will be a crucial part [23]. He separates a body of policy-oriented scholarship on sustainability transitions which is generally concerned with the dynamics of structural change and, specifically, how to intervene through disruption of existing systems (e.g., [24] or through alignment of different actors attempting to govern the process of change (e.g., [25]). In contrast, Swilling identifies a body of scholarship for which a sustainability transition entails challenging dominant institutions and practices (see also [26]) but argues that despite its promise, such a body of scholarship is limited in understanding how such a fundamental change can happen in practice. Swilling argues for approaches capable of apprehending the multiple changes while simultaneously engaging with forms of ‘radical incrementalism’ [23]. The promise of such radical incrementalism is that it could help apprehend the impact of multiple changes in experimental zones [15]. Yet, scholars of sustainability transitions have pointed to the difficulty of moving from experimental theories to broader processes of structural change, beyond often simplistic ideas of scaling up (for a multi-perspectival critique see: [27]).

These concerns generate a temptation to replicate similar questions to those who have long guided the literature on sustainability transitions and emphasized the synchronicity and alignment of different actors and interests in transitions. This literature on sustainability transitions has been applied in multiple contexts. Still, it was developed concerning empirical examples, first in the Netherlands, then in other Western European countries and North America [28]. Often, this literature struggles to explain change processes beyond the contexts in which they were developed [29]. While the long-term perspective adopted in transitions theory and its engagement with the material aspects of change finds resonance across different contexts (for example, Swilling [23] uses it effectively), there is a risk involved in an uncritical adoption of extraneous theory across contexts. The extent to which social change can be examined through a transitions lens is increasingly questioned as part of a broader colonizing discourse that establishes global normative ideals based on systems of prioritization that are alien to those contexts in which they have to be deployed.

Looking at a diversity of geographical contexts can help rethink the epistemological and ontological assumptions that form the foundation of transition theories. Recently, calls have been made to consider spatial disparities in energy transitions on a global or national scale [30]. Thinking of transitions in contexts already facing significant gaps in service provision is particularly intertwined with questions of justice. It requires a sophisticated approximation to the social dynamics and politics of transition that move beyond traditional conceptualizations of the energy transition in the context of development as a question of capacity building or technology transfer [31]. In Africa, colonial histories bear a particular weight in the structure of discourses, policies, and infrastructures that shape energy transitions [32].

This special issue explores the governance of energy transitions in Africa without taking any theoretical approaches to understanding socio-technical transition for granted. Instead, our objective at the outset was to capture a wide range of approaches to understanding the transition, emphasizing the importance of social factors beyond complex modelization or theoretical structuration of the transition process [33]. The objective, instead, was to reflect on the plurality of context of transitions in Africa alongside a plurality of perspectives. Our purpose is to develop understandings of sustainability transitions that do not take for granted its explanation, but that depart instead from engagements with the diversity of changes that aggregate into transition pathways—a diversity that in the context of Africa is impossible to overlook. This special issue follows an international workshop held in Ghana in September 2019. Participants were asked to engage with the topic of the governance of energy transitions both empirically, with material from Africa, and conceptually. The discussions at the workshop focused on three key themes for future research, for which the papers in this article collection provide an introduction:

1. Transition dynamics are highly context-sensitive and place-specific. Our theorization of transitions needs to be built upon such place-based specificities, as well as global interdependencies and dynamics between different geographical scales.
2. Colonial pasts often shape current investments in energy infrastructures, e.g., via knowledge production or developmental agendas, with influential roles played by donor agencies, development banks, and trans-national (state-owned) enterprises. This calls for a differentiated and historically informed analysis of the political economy of energy investments.
3. Furthermore, notions of energy justice and democracy relate to questions of the provision of basic needs, the relationship between energy transitions, energy crisis, and energy access, and what adverse effects of energy infrastructures particular groups reasonably have to accept. Often, notions of justice are intertwined with complex ideas of epistemic justice [34], for example, when individuals are excluded from decision-making. By empirically researching justice concerns and democratic aspirations of individuals, and by giving voice especially to ‘silenced voices,’ researchers can contribute to articulating alternatives and support productive ways of dealing with conflicts.

These themes are further explored in the three "Introduction", "Understanding the specificity of interdependent, yet place-based transitions", "Engaging with history and the political economy of energy transitions" sections below. Based on that, we reflect on more plural understandings of societal change in "Appreciating plural notions of energy justice" sections; before we bring a final reflection on the genesis and outcome of this article collection in "Conclusion" sections.

### Understanding the specificity of interdependent, yet place-based transitions

Sustainable Energy Transitions (SETs) are tied to mundane aspects of everyday life for the one billion people who lack access to electricity and the three billion who lack access to clean fuels. Complex sustainability innovations overlap with low-tech alternatives in a material and technological context characterized by heterogeneity. New ways of understanding SETs through thermal energy technologies for cooking, solar home systems, or electrification strategies have become increasingly crucial for most of the world, for which renewable transitions go hand in hand with energy access challenges. After outlining “justice pitfalls of urgent energy transitions in the global South,” Kumar et al. ask: “how do we bring together these two ideas—one that calls for abstract universal unity and the other for the recognition of individual needs and differences?” ([35]; p. 8). A globally homogenized view on ‘the’ energy transition that differentiates between a standard ‘Global South’ and a standard ‘Global North’ transition misrepresents both the geography of transitions and their dynamics.

First, there is no standard energy transition because energy infrastructure has always been organized in heterogeneous ways [36]. Systems vary across geographies, and multiple systems of infrastructure coexist within a given city or region [37]. Heterogeneity also shapes everyday practices and citizenships as infrastructure users adjust and adapt to existing infrastructure configurations [38]. Moreover, transitions anywhere are embedded in broader economic and political contexts. Essential factors like world markets, international governance networks, and their ideas are present globally but can still be more or less effective in different settings. Innovation ideas circulate across contexts, while the broader economic and cultural dynamics in which transition occurs are shaped beyond any single geography (see the next section). Innovation practices are also determined in regional, multi-national clusters of countries. Heterogeneous contexts with heterogeneous histories have thus produced myriad socio-technical pathways and material infrastructures that constitute complex energy landscapes. Most of the research done in Africa strongly supports this proposition.

In their contribution to this collection, Koepke et al. (2022), for example, show how "socio-technical heterogeneity contributes significantly to the functioning of Southern cities by responding to user demands that are unmet by conventional, centralized grids" e.g. when "socio-technical alternatives" in electricity services "serve low-income users, the middle classes, and urban elites".

In line with this heterogeneous reality of energy landscapes, Edomah’s (2022) paper in this article collection leans on various epochs to contextualize the energy transition and energy systems change in Nigeria: pre-industrial (1800); early industrial (1850); industrial (1900); late industrial (1950); and information (2000). Accordingly, ‘… from the preindustrial (agricultural) era … the 1800s, we notice a gradual change in technology use and social practices that became more energy intensive, thus requiring more energy dense sources…’ (Edomah, 2022).

Berté and Adou (2022, this collection) report a similar socio-technical trajectory for Côte d'Ivoire. However, in their study, they mainly focus on the micro-dynamics of energy choice taking the bakery sector in Abidjan as an example. With its strong dependence on wood as a source of heating energy, bakeries are a main contributor to forest degradation and deforestation in Côte d'Ivoire. Alternative sources of energy have been propagated for long, however, without resounding success so far. Applying a social practice theory approach, Berté and Adou identify different driving factors. First, and very much in line with the more policy-oriented literature in the field, the authors describe the governance of energy in Côte d’Ivoire as a “highly fragmented regulatory space”, which hampers the effective implementation of policies. But there are more context-specific factors that only an in-depth case study can unearth: Among others, the authors report about the absence of civil society organizations that might push for a transition, economic constraints that set tight limits to feasible business models of bakeries, and path-dependencies in the development of infrastructure.

Building on the case of deployment of digitalization technologies in Nigeria and South Africa, Nawaiwu (2022, this collection) explores the potential of digital technologies in energy transitions. The article sets up the following argument in favor of digital technologies:

“*The use of digital technologies to enable sustainable energy transitions involves the adoption and implementation of these classes of technologies in ways that leverage their unique characteristics to offer new models of production, distribution, and consumption of energy*.”

In this context, among the promises of digital technologies is the possibility to move households away from the generation of energy, most often from fossil fuel sources, and to let them join platforms through which renewable energies can be implemented, for example, through the constitution of smart grids or the use of blockchain technology. The results show an apparent disconnect between the regulatory and policy environment in which such technologies are deployed, on one hand, and the localized efforts to implement them on the other. A similar concern informs the analysis of Gebresslassie et al. (2022, in this collection), in which regulatory environments are not always open to the adoption of challenging technologies, in this case, decentralized energy systems.

Their papers reflect on the heterogeneous co-construction of socio-technical relations in processes that simultaneously sediment material infrastructures and governance structures. This explains the stubborn persistence of the socio-technical systems that structure our life—what Hommels called obduracy [39]. Perhaps the next generation of research on transitions can also analyse how intentions of change, and the resulting changes, can be integrated within such systems in the forms of localized, experimental interventions.

### Engaging with history and the political economy of energy transitions

Energy transitions are shaped by complex histories of colonial and imperial domination, which become highly visible in contexts where such histories are recent or even still present. Newell [40] has argued that energy transitions must engage with the racialized nature of energy systems, as manifested in the material constitution of infrastructures, the governance of energy systems, and their operation. The constitution of energy systems placed value on some lives over others, and transition efforts so far have done little to address the racialized constitution of energy systems [41].

For example, to the extent that energy transitions are connected to decreasing global GHG emissions, there is a danger that they become an arena for ‘carbon colonialism.’ A common understanding of carbon colonialism relates to the concern that powerful countries (in the ‘North’) invest and coerce other countries to occupy the ‘discursive and physical spaces in the global South’ in the name of environmental and climate protection while also masking historical responsibilities in accounting for carbon emissions ([35]; p. 5). They say:

“*Putting a price on and commodifying emission responsibilities means that historical injustices of extraction and colonization, which have led to inequalities of wealth between (as well as within) the global North and South, can continue in new forms*” ([35]; p. 6)

Yet, it is not only a question of the reproduction of the imperial structures of colonialism across transnational spaces. Colonial histories are already inscribed in the spaces of transition and shape not only what practices are possible but also what futures are imaginable [32]. The articles in this collection demonstrate the complex interplay between global pressures on the energy transition and place-based dynamics.

For example, Koepke et al. (2022, in this collection) observed in Dar es Salaam that "development partners have promoted managerial modalities to reform African infrastructure sectors whose state-led, hierarchical organization was considered inefficient.” Such past interventions seem to have contributed significantly to the infrastructural heterogeneity and to the institutional complexity seen today, which make it particularly difficult to orchestrate energy transitions. The tension between diversity in initiatives and the need to align those with evidence of the actual occurrence of a transition is visible in the analysis.

Edomah’s (2022) paper in this collection also acknowledges the role of historical antecedents, and political and economic influences on Nigeria's energy transition and energy systems change. Historically and politically, the paper points to three agents in the Nigerian electricity sector that have impacted directly on energy transition and energy systems change: changing perceptions and goals (1890–the 1960s); direct government interventions (1940s–1970s); and changes in market rules (2005 onwards). First and foremost, in the era before independence, the colonialists determined their energy needs, which led to the importation of their choice of power plants, culminating in the tinkering with various fuel sources and technologies and the creation of lock-ins (pg. 6). Secondly, direct government intervention from the 1940s facilitated a somewhat increased development of electricity infrastructure and access to consumers. The liberalization of the electricity market from 2005 onwards triggered the creation of important policies and agencies, thereby empowering political authorities to influence the infrastructure development decisions of the energy landscape.

Historical trajectories also shaped the possibilities of implementing innovative, decentralized energy systems in Ethiopia and Mozambique (Gebreslassie et al., 2022, this collection). The colonial history of Mozambique weights heavily on the organization of the electricity system, and electricity provision is still considered part of the political toolkit of the government FRELIMO. In Ethiopia, with no colonial past, the question is the alignment of electricity provision objectives with notions of nationhood, strongly linked, for example, with large dams and hydropower production. In both cases, electricity provision ideals are complexly intertwined with the construction of nationhood, making it difficult even to imagine decentralized alternatives, which are often discarded as not being sufficiently modern. In both cases, however, changing political alliances in energy provision and pressure from diverse sectors have led to nationwide programmes for promoting decentralized energy (including a new off-grid development regulation in the case of Mozambique).

Osei-Tutu et al. (2021, in this collection) explore how international capital flows shape Ghana’s transition. They report about market trends related to the electricity sector in Ghana. One of those trends is a shift from an exclusive state supply to a state-private supply mix, with international investors playing a prominent role: For capital-intensive hydroelectricity projects, the Ghanaian government has brought on board Chinese companies; for the time-critical fixing of problems with prolonged blackouts, the government entered into a 10-year contract with a Turkish energy group to provide thermal electricity from two floating power ships. All these financing arrangements, mainly due to the substantive amounts and related contractual intricacies, create further dependencies and close sustainable development options for years to come.

A critical difference in the factors that influence energy policy in primarily colonized (rather than colonizing) areas of the world is the role that international agencies and networks have on development agendas and how infrastructures are financed and developed. There is an apparent concern about how international development agendas and donor agencies shape SETs. Over the last few years, more and more empirical research has been conducted to reveal the trans-national interplays that make energy politics much more complex, moving beyond the methodological nationalism that has shaped much research on the energy transition described above. The papers in this special issue engage with the dynamics between national policy, investment, and international capital flows, but there is room for a better understanding of the place-based dynamics of change that shape Africa’s governance today. Studies now start to more comprehensively chart the diversity of actors that shape energy transitions [42, 43], and new theorizations of such interdependent governance are likely to develop.

### Appreciating plural notions of energy justice

The quest for appreciating diversity is undoubtedly most important when understanding notions of justice, which can differ not only from one person to the next but also from one moment to the other.

Energy transitions are happening in a context of increasing attention to the distributional impacts of extensive infrastructure reconfigurations. While in the context of energy, a sustainability transition is linked to the remediation of energy access challenges, in practice, energy transitions will directly impact different population groups. Recent work has put notions of energy justice at the forefront [44]. Following scholarship on environmental justice, ideas of energy justice emphasize distributional aspects of socio-technical transitions, the recognition of a diverse set of needs and impacts, and the representation and participation of a wide range of stakeholders in the politics of energy.

While questions of justice are already integrated into transitions scholarship [45, 46], they are even more salient in transitions unfolding in the context of significant disparities of income and infrastructure access across the population and alongside complex energy access challenges. As Swilling argues, it is unlikely that the energy transition will be just [23] if it can happen at all:

“…the expected just transition this could give rise to will not happen simply because there is a shared normative commitment, as is now reflected in the adoption of the SDGs [Sustainable Development Goals] and before that in the GED [Green Economy Discourse]. Nor will much progress be made by formulating bland managerial policy prescriptions that ignore underlying power dynamics and paradigm differences.” ([47]; p. 667)

The complexity of such constellations cannot be overemphasized. Koepe et al. (2022, in this collection), for example, show how managers at Dar es Salaam’s integrated electricity utility TANESCO struggle to navigate across different modes of governance to accommodate “conflicting managerial interests and hierarchically set goals while justifying this practice as serving broader public interests.”

In the context of digitalization in Nigeria and South Africa, Nwaiwu (2022, this collection) recovers the leapfrogging argument, demonstrating that digitalization offers a new leapfrogging promise:

“What can be learned from the current state of sustainable energy transitions in Nigeria and South Africa is that digital technologies offer the possibility of a more efficient way to leapfrog the infrastructure deficit required to address energy poverty in sub-Saharan Africa.”

The challenge, as the paper explains, is that it is still too early to understand the distributional impacts of digital technologies, although they can facilitate small-scale electricity generation (mini-grids). Transparent billing (facilitated by blockchains) may simply curtail access to technology, further reinforcing distributive and procedural injustices.

Just transition’ claims relate not only to the need to examine the impacts of those transitions but also point toward the need to link transitions to broader drivers of structural oppression and exclusion that shape people’s lives. Intersectional approaches that seek to redress existing injustices are increasingly central to the energy transition, and the experiences from Africa are putting those approaches at the forefront.

### Origin and purpose of this article collection

We started our endeavor by recognizing a “global Northern bias” in how energy transitions are understood globally. From our own experiences, we suspect that there is, in fact a “global Northern narrative,” i.e. “particular imaginaries of what ‘appropriate’ and ‘adequate’ energy have come to mean for researchers and practitioners in the global North […] [T]hese imaginaries and meanings permeate (often through advocacy from countries in the North) energy policy and discourse in the global South” ([35]; p. 7). Suspicion of Western-led models of thought and policy, even when wrapped in energy justice narratives, is highly suspicious, even referred to as ‘energy bullying’ [48]. Again, without too much homogenization, we can think of ‘epistemic worlds’ that are separate from each other, and some of them tend to dominate globally. Hence, it is probably worth searching for those frequently overlooked epistemologies [34].

For example, examining energy transitions requires a parallel critical examination of the extent to which particular ideas of ‘development’ and ‘market logic’ are engrained into widespread expectations on how energy transitions must be brought about and unfold. For example, Kumar notes “the prominent place that non-state actors have taken in the provision of energy, especially electricity, in the last decade” ([35]; p. 7). More fundamentally, most scholars studying socio-technical transitions—often unconsciously—buy into a quasi-evolutionary understanding of socio-technical change and consequently search for and promote niches in which technologies can be nurtured to bring about much desired societal change. Alternatives to this approach are imaginable yet hardly found in the literature. How do conceptualizations of societal change or socio-technical (sustainability) transitions support an understanding of transitions in less known geographies? In analogy to the caution that is due when applying the general notion of “sustainability transitions” to geographies different from Western Europe, where the concepts have been developed [49], we question whether old heterodoxies about SETs need not be merged with new ideas. Should the analysis of SETs in Africa, for example, be built on an entirely different transition vocabulary? Who can free themselves from the biases that these approaches bring with them, such as an over-emphasis on the role of technology and innovation in societal change? Can greater attention to informal rules and networks improve our understanding of energy landscapes globally? A search for more diverse epistemologies that (may) have emerged in communities that are rather underrepresented in academic mainstream debates promises to be productive here.

Driven by the quest to foreground the contextualization of energy transitions discourse on the global south, from 15 to 17th September 2019, fourteen scholars from eleven countries convened at the Campus of the University of Ghana, Legon, in Accra, to discuss the governance of energy systems in Africa. The presentations covered a broad range of regional contexts with case studies mainly from West Africa (Ghana, Nigeria, Benin, Côte d'Ivoire) but also from East Africa (Uganda, Tanzania, Kenya) and South Africa. They also drew on a wealth of different theoretical perspectives. However, the presentations shared striking commonalities, too. First, there was the emphasis on complex governance processes influenced by diverse actors, not only within national boundaries but often far beyond (e.g. US or European donor agencies or state-owned enterprises from China). The second thread of discussions addressed the often unanticipated ways in which everyday practices (e.g., in households or small enterprises) respond to changing energy policies. In contrast to the widespread expectation that energy systems could be governed comprehensively and systematically, the empirical insights discussed at the workshop illustrated the multiple ways in which long-term strategies are usually superimposed and constrained by more ad-hoc and partly unintended dynamics. This certainly not only holds for African contexts, but the workshop demonstrated that there are distinct regional patterns in governance arrangements, and some of these distinct realities are not yet adequately reflected in scholarly analyses of energy governance.

The initial enthusiasm of the participants to be part of this article collection, however, did not translate into full publications in all cases. Several scholars from this workshop could not participate in this endeavour. Reflecting on the process, we find at least three reasons that have impeded the scholars from finally publishing their contributions.

First, there was a question of academic languages, whether because some of the papers were written in other languages or with the jargon of different disciplines, or whether they did not meet the academic requirements of publication, for example, in terms of presenting the methodology and empirical materials. Many of the papers in the workshop were qualitative in nature because of the exploratory character of transitions research in Africa. Many were also built on a wealth of experience and exchanges but not necessarily codified in interviews or other methodological materials. And while the energy transition is undoubtedly a transdisciplinary question, disciplines like anthropology were not easily accommodated in the collection.

Second, there were questions about how the timing of the special issue aligned with the career of the scholars that participated in the workshop. Most of the scholars we invited were in the late stages of their Ph.D. or starting a post-PhD job. Thus, many saw their careers changing rapidly following the workshop. In Africa, there is currently a shortage of skilled applicants in the energy sector, so some of the participants in the workshop quickly found non-academic and well-paid jobs in the energy sector, mainly doing consultancy and other knowledge-intensive roles. A few scholars started careers in academia and were called to teach. In summary, the moment this workshop took place and the strict labor demands of the African context meant that the scholars did not have the time to dedicate to the article in the post-workshop period.

Finally, some scholars highlighted the limited mentorship and support they have received in their institutions or networks. For many participants, their interest in energy transitions is cultivated in isolation, without a responding voice that can engage with the specificity of their research. During the workshop and subsequent months, we tried to provide support, but as a team, we were also limited and even more limited during the COVID-19 pandemic. However, the experience demonstrates the enduring influence of networks such as the one we tried to form at the University of Ghana and moves us to commit to further efforts to create networks studying the energy transition in Africa and elsewhere.

## Conclusion

This collection offers a sketch for a research agenda on transitions research in Africa, one whose contours are still ill-defined, but which engages with the central themes of heterogeneity, politics, and the material basis of energy transitions. The collection does not abandon the methodological nationalism that has permeated most energy transitions research in Africa. Still, it points towards a diversity of perspectives and entry points that may constitute this agenda.

In the time we developed this special issue the scholarship on decolonial perspectives on energy transitions have grown. Decolonization means engaging with the challenges of coloniality—the pervasive persistence of systems of thought inherited from the colonization process [50]. Energy transitions, as deliberate efforts to catalyze social change, also imply challenges to established forms of thinking—in the case of post-colonial African nations, transitions must challenge those understandings of energy needs and energy provision inherited from the European colonizers [51]. The energy transition can only hence be a decolonizing one.

This is an urgent plan but one that remains underdeveloped and worse off, dominated by international interests whose dominant narratives are increasingly seen as threatening by a research community increasingly engaged in understanding the distributional impacts of energy transitions.

## Data Availability

Data sharing not applicable to this article as no datasets were generated or analysed during the current study.
